# Intraperitoneal Exposure to Nano/Microparticles of Fullerene (C_60_) Increases Acetylcholinesterase Activity and Lipid Peroxidation in Adult Zebrafish (*Danio rerio*) Brain

**DOI:** 10.1155/2013/623789

**Published:** 2013-06-20

**Authors:** Gonzalo Ogliari Dal Forno, Luiza Wilges Kist, Mariana Barbieri de Azevedo, Rachel Seemann Fritsch, Talita Carneiro Brandão Pereira, Roberta Socoowski Britto, Sílvia Stanisçuaski Guterres, Irene Clemes Külkamp-Guerreiro, Carla Denise Bonan, José María Monserrat, Maurício Reis Bogo

**Affiliations:** ^1^Laboratório de Biologia Genômica e Molecular, Faculdade de Biociências, Pontifícia Universidade Católica do Rio Grande do Sul, Avenida Ipiranga 6681 Sala 134, 90619-900 Porto Alegre, RS, Brazil; ^2^Instituto Nacional de Ciência e Tecnologia Translacional em Medicina (INCT-TM), 90035-003 Porto Alegre, RS, Brazil; ^3^Universidade Federal do Rio Grande-FURG, Instituto de Ciências Biológicas (ICB), Avenida Itália Km 8 s/n, 96208-900 Rio Grande, RS, Brazil; ^4^Programa de Pós-Graduação em Ciências Fisiológicas-Fisiologia Animal Comparada, FURG, Avenida Itália Km 8 s/n, 96208-900 Rio Grande, RS, Brazil; ^5^Faculdade de Farmácia, Universidade Federal do Rio Grande do Sul, Avenida Ipiranga 2752, 90610-000 Porto Alegre, RS, Brazil; ^6^Laboratório de Neuroquímica e Psicofarmacologia, Faculdade de Biociências, Pontifícia Universidade Católica do Rio Grande do Sul, Avenida Ipiranga 6681, 90619-900 Porto Alegre, RS, Brazil; ^7^Instituto Nacional de Ciência e Tecnologia de Nanomateriais de Carbono, 31270-901 Belo Horizonte, MG, Brazil

## Abstract

Even though technologies involving nano/microparticles have great potential, it is crucial to determine possible toxicity of these technological products before extensive use. Fullerenes C_60_ are nanomaterials with unique physicochemical and biological properties that are important for the development of many technological applications. The aim of this study was to evaluate the consequences of nonphotoexcited fullerene C_60_ exposure in brain acetylcholinesterase expression and activity, antioxidant responses, and oxidative damage using adult zebrafish as an animal model. None of the doses tested (7.5, 15, and 30 mg/kg) altered AChE activity, antioxidant responses, and oxidative damage when zebrafish were exposed to nonphotoexcited C_60_ nano/microparticles during 6 and 12 hours. However, adult zebrafish exposed to the 30 mg/kg dose for 24 hours have shown enhanced AChE activity and augmented lipid peroxidation (TBARS assays) in brain. In addition, the up-regulation of brain AChE activity was neither related to the transcriptional control (RT-qPCR analysis) nor to the direct action of nonphotoexcited C_60_ nano/microparticles on the protein (*in vitro* results) but probably involved a posttranscriptional or posttranslational modulation of this enzymatic activity. Taken together these findings provided further evidence of toxic effects on brain after C_60_ exposure.

## 1. Introduction

Nanoscience investigates nanoscale phenomena and serves as the foundation for nanotechnology, which develops practical applications for nanomaterials (particles typically with sizes in the 1–100 nm range but not exclusively) [1]. Due to their composition, small size, and shape, nanomaterials exhibit novel properties for diverse applications that have already yielded in a variety of commercially available products [2]. As a consequence, it is expected that both humans and environmental systems will be increasingly exposed to nanomaterials in the next years. Nanotoxicology [3] and nanoecotoxicology [4] are emerging disciplines that arose to address the potential human and environmental health effects of nanomaterials' exposure.

Fullerenes are molecular materials that call attention after the first preparation of C_60_ which is an allotrope of carbon consisting of 60 carbon atoms joined to form a cage-like structure [5]. C_60_ exhibits unique physical and chemical properties for many technological applications, such as electronics, superconductors, cosmetics [6], and, more recently, drug and gene delivery [7]. Although the toxic effects of C_60_ are still mostly unknown, especially those related to neurotoxicity, some general aspects have already been addressed. For instance, C_60_ is reported to be cytotoxic to various mammalian cells [8] and it has been shown to induce lipid peroxidation in human cells [9], in brain of juvenile largemouth bass (*Micropterus salmoides*) [10], and in common carp (*Cyprinus carpio*) [11]. Moreover, C_60_ was classified as “very toxic” to aquatic organisms by the grid for the potential ecotoxicological hazard evaluation which is based mainly on toxicity to fish, *Daphnia*, and algae and in data about degradability of the substance [4].

In cholinergic neurotransmission, choline acetyltransferase (ChAT) is responsible for the synthesis of acetylcholine (ACh) in the presynaptic neuron. After its release into the synaptic cleft, ACh promotes the activation of metabotropic muscarinic and ionotropic nicotinic cholinergic receptors. The reaction responsible for the maintenance of levels of ACh is catalyzed by two cholinesterases (ChE): acetylcholinesterase (AChE) (E.C. 3.1.1.7) and butyrylcholinesterase (BuChE) (E.C. 3.1.1.8) [12]. The zebrafish (*Danio rerio*) has long been considered a powerful animal model for studying several biological events. More recently, zebrafish become also a valuable model to environmental and toxicological studies [13]. It has been demonstrated that BuChE is not encoded in the zebrafish genome, but AChE is encoded by a single gene that has been cloned, sequenced, and functionally detected in zebrafish brain [14].

AChE activity has been widely used as a bioindicator of environmental exposure to neurotoxic pollutants. For example, the inhibition of AChE as a biomarker for assessment of the exposure of organisms to organophosphate and carbamate insecticides is well known [15]. The inhibition of brain AChE activity of aquatic species by toxic substances such as methanol [16], heavy metals mercury, and lead [17], neurotoxins [18], and organochlorine pesticide endosulfan [19] also has been well established. On the other hand, AChE activation has also been demonstrated as a consequence of exposure to neurotoxic compounds such as aluminum [20] and ethanol [21]. 

Therefore, considering that (i) both humans and environmental systems will be increasingly exposed to C_60_ in a near future, (ii) the neurotoxic effects of C_60_ are far from being completely understood, (iii) measurement of AChE activity in organisms is used as a biomarker of environmental contamination due to neurotoxic substances, (iv) determination of oxidative stress parameters is recognized as a tool to evaluate toxicity mediated by small particles exposure, and (v) zebrafish is a well-established organism for toxicological analysis, the aim of the present study was to evaluate the nonphotoexcited C_60_ effects in brain AChE activity and its gene expression pattern. Furthermore, we have analyzed the effects of the nonphotoexcited C_60_ in parameters related to antioxidant defenses and lipid peroxidation in the brain of zebrafish.

## 2. Materials and Methods

### 2.1. Chemicals

Fullerene (C_60_, 99.5% purity) was purchased from Aldrich (Milwaukee, WI, USA); DMSO was purchased from Fisher Scientific (Pittsburgh, PA, USA) and Trizma Base; ethylenediaminetetraacetic acid (EDTA), ethylene glycol bis (beta aminoethylether)-N,N,N′,N′-tetraacetic acid (EGTA), sodium citrate, Coomassie Blue G, bovine serum albumin, acetylthiocholine, 5,5′-dithiobis-2-nitrobenzoic acid (DTNB) HEPES, BHT (99%), 2,2′-azobis(2-methylpropionamidine) dihydrochloride (ABAP), and 1,1,3,3-tetramethoxypropane were purchased from Sigma Chemical Co. (St. Louis, MO, USA). KCl and SDS (90%) were purchased from Labsynth (Brazil). Tetramethoxypropane (TMP) and 2′,7′-dichlorodihydrofluorescein diacetate were purchased from Acros Organics (Morris Plains, NJ, USA) and Molecular Probes Inc. (Eugene, OR, USA) respectively. MgCl_2_ and Acetic acid 99.7% were purchased from Isofar and Vetec (Brazil), respectively. TRIzol reagent, Platinum Taq DNA Polymerase, and SYBR Green I were purchased from Invitrogen (Carlsbad, CA, USA). ImProm-II Reverse Transcription System was purchased from Promega (Madison, WI, USA). All other reagents used were of analytical grade.

### 2.2. Animals

Adult wild-type zebrafish (*Danio rerio*, Cyprinidae) of both sexes (6–9 months old) were obtained from a specialized supplier (Redfish Agroloja, RS, Brazil). Animals were kept at a density of up to five animals per liter in 50 L housing tanks with tap water that was previously treated with Tetra's AquaSafe (to neutralize chlorine, chloramines, and heavy metals present in the water that could be harmful to fish) and continuously aerated (7.20 mg O_2_/L) at 26 ± 2°C, under a 14/10 h light/dark controlled photoperiod. Animals were acclimated for at least two weeks before the experiments and were fed three times a day with TetraMin Tropical Flake fish food. The fish were maintained healthy and free of any signs of disease and were used according to the Guide for the Care and Use of Laboratory Animalspublished by the US National Institutes of Health. All procedures in the present study were approved by the Animal Ethics Committee of the Pontifical Catholic University of Rio Grande do Sul (PUCRS), protocol number 10/00185-CEUA.

### 2.3. C_60_ Suspension

Suspensions of C_60_ in DMSO were prepared as previously described [23, 24] with modifications. Briefly, 7.6 mg of C_60_ was added to 0.5 mL of DMSO and sonicated for 3 h. The concentrated suspension of C_60_ obtained was diluted in water to result in suspensions that when intraperitoneally injected (mean injection volume was 10 *μ*L) get the doses of 7.5, 15, and 30 mg/kg fish (12.5% DMSO). For this reason, the C_60_ suspensions were named as 7.5, 15, and 30. The doses and times of exposure were selected based on previous studies where multiwalled carbon nanotubes were intravenously injected in mice [25, 26], and functionalized single-walled carbon nanotubes were intraperitoneally injected in rats [27]. C_60_ suspensions were further sonicated for one hour prior to use. To avoid C_60_'s photoexcitation, the C_60_ suspensions were prepared and stored in a dark condition.

### 2.4. Characterization of C_60_ Suspensions

The C_60_ suspensions (7.5, 15, and 30) were characterized in terms of particle size distribution [28]. The mean diameter over the volume and number distribution (*d*_4.3_) was determined by laser diffractometry (Mastersizer 2000, Malvern Instruments, UK). The value of SPAN was utilized to determine particle size distribution according to (1), where *d*_0.9_, *d*_0.1_, and *d*_0.5_ are the particle diameters determined at 90%, 10%, and 50% cumulative undersized volumes, respectively,

(1) $$\begin{array}{c} \text{SPAN}\;=\frac{d_{0.9}-d_{0.1}}{d_{0.5}}. \end{array}$$

### 2.5. Animal Procedures

Intraperitoneal (i. p.) injection was adopted as administration route in the *in vivo* protocols (i) to avoid the photoexcitation of the C_60_ material and (ii) to ensure that exposure concentrations are in line with target values. Intraperitoneal injections were conducted using a 3/10-mL U-100 BD ultra-fine short insulin syringe 8 mm (5/16′′) × 31G short veedle (Becton Dickinson and Company, NJ, USA) according to the protocol established by Phelps and colleagues [29]. Briefly, the volume injected into the animal (mean injection volume was 10 *μ*L) was adjusted to the fish bodyweight (mean mass of the animals was 0.75 g ± 0.06 g/mean ± SEM) to achieve 7.5, 15, and 30 mg/kg based on a previous study [30]. The animals of the control group received the same volume of saline solution, and the animals of the vehicle control received the same volume of 12.5% DMSO. Anesthesia of the animals prior to the injection was obtained by its immersion in a solution of benzocaine (1 mM in MeOH 1%) until the animal showed a lack of motor coordination and reduced respiratory rate. The anesthetized animal was gently placed in a water-soaked gauze-wrapped hemostat with the abdomen facing up and the head of the fish positioned at the hinge of the hemostat (the pectoral fins were used as a landmark on the abdomen). The needle was inserted parallel to the spine in the midline of the abdomen posterior to the pectoral fins. The injection procedure was conducted in such a way as to guarantee that the animal did not spend more than 10 s out of the water. After the injection, the animals were placed in a separate tank with highly aerated nonchlorinated tap water (25 ± 2°C) to facilitate recovery from the anesthesia. Saline solution was used as control. All the animals that recovered within 2-3 min following the injection continued in the experiment, while animals that did not recover during this period were discarded. Six, twelve, or twenty-four hours after the injection, the animals were euthanized. 

### 2.6. *In Vitro* Assays of AChE Activity

*In vitro* assays were performed as previously described [31, 32] in order to evaluate if 7.5, 15, and 30 C_60_ suspensions might have a direct effect on the enzyme. Briefly, 33 *μ*L of C_60_ suspensions were added to the reaction medium before the preincubation with the enzyme-containing lysate from zebrafish brain homogenate and maintained during the enzyme assays. Control treatments with equal volume of vehicle (DMSO 12.5%) were performed to exclude the DMSO effect on the enzyme activities.

### 2.7. Determination of AChE Activity

Zebrafish were euthanized, and their whole brains were removed by dissection. The brains (two whole brains for each sample) were homogenized on ice in 60 volumes (v/w) of Tris-citrate buffer (50 mM Tris, 2 mM EDTA, 2 mM EGTA, pH 7.4, adjusted with citric acid), in a glass-Teflon homogenizer. The rate of acetylthiocholine hydrolysis (ACSCh, 0.88 mM) was assessed in a final volume of 300 *μ*L with 11 mM phosphate buffer, pH 7.5, and 0.22 mM DTNB using a method previously described [33]. Before the addition of substrate, samples containing protein (5 *μ*g) and the reaction medium described above were preincubated for 10 min at 25°C. The hydrolysis of substrate was monitored by the formation of thiolate dianion of DTNB at 412 nm for 2-3 min (intervals of 30 s) in a microplate reader. Controls without the homogenate preparation were performed in order to determine the nonenzymatic hydrolysis of the substrate. The linearity of absorbance against time and protein concentration was previously determined. The AChE activity was expressed as micromoles of thiocholine (SCh) released per hour per milligram of protein. All enzyme assays were evaluated in triplicate, and at least four independent experiments were performed.

### 2.8. Gene Expression Analysis by Quantitative Real-Time RT-PCR (RT-qPCR)

Gene expression analysis was carried out only when kinetic alteration occurred. For this reason, immediately after 24 hours of intraperitoneal injection (C_60_ suspension 30), the animals were euthanized by decapitation. For each sample, a pool of three zebrafish whole brains was used. Total RNA was isolated with Trizol reagent (Invitrogen, Carlsbad, CA, USA) in accordance with the manufacturer's instructions. The total RNA was quantified by spectrophotometry and the cDNA was synthesized with ImProm-II Reverse Transcription System (Promega) from 1 *μ*g total RNA, following the manufacturer's instructions. Quantitative PCR was performed using SYBR Green I (Invitrogen) to detect double-strand cDNA synthesis. Reactions were done in a volume of 25 *μ*L using 12.5 *μ*L of diluted cDNA (1 : 100 for *EF1*α** and *Rlp13*α**; and 1 : 20 for *ache*), containing a final concentration of 0.2x SYBR Green I (Invitrogen), 100 *μ*M dNTP, 1x PCR Buffer, 3 mM MgCl_2_, 0.25 U Platinum Taq DNA Polymerase (Invitrogen), and 200 nM of each reverse and forward primers (Table 1). The PCR cycling conditions were an initial polymerase activation step for 5 min at 95°C, 40 cycles of 15 s at 95°C for denaturation, 35 s at 60°C for annealing, and 15 s at 72°C for elongation. At the end of cycling protocol, a melting curve analysis was included and fluorescence measured from 60 to 99°C. Relative expression levels were determined with 7500 Fast Real-Time System Sequence Detection Software v.2.0.5 (Applied Biosystems). The efficiency per sample was calculated using LinRegPCR 11.0 Software (http://LinRegPCR.nl) and the stability of the references genes, *EF1*α** and *Rlp13*α* (M-value),* and the optimal number of reference genes according to the pairwise variation (*V*) were analyzed by GeNorm 3.5 Software (http://medgen.ugent.be/genorm/). Relative RNA expression levels were determined using the 2^−ΔΔCT^ method.

### 2.9. Antioxidant Capacity against Peroxyl Radicals

Total antioxidant capacity against peroxyl radicals was performed according Amado and colleagues [34], employing the thermal decomposition of 2,2′-azobis 2 methylpropionamidine dihydrochloride (ABAP; 4 mM) as peroxyl radical generator. Reactive oxygen species (ROS) concentration was detected with the fluorescent probe 2′,7′-dichlorofluorescein diacetate (H_2_DCF-DA) in a final concentration of 40 *μ*M (480 and 525 nm forexcitation and emission, resp.). The relative difference between ROS area with and without ABAP was considered a measure of antioxidant capacity, with high area difference meaning low antioxidant capacity, since high fluorescence levels were obtaining after adding ABAP, meaning low competence to neutralize peroxyl radicals.

### 2.10. Measurement of Lipid Peroxidation

Lipid peroxidation was measured through the determination of thiobarbituric acid reactive substances (TBARS), following the methodology of Oakes and van der Kraak [35]. Brain homogenates (10 *μ*L) were added to a reaction mixture made with 150 *μ*L of 20% acetic acid, 150 *μ*L of thiobarbituric acid (2.4%), 50 *μ*L of Milli-Q water and 20 *μ*L of sodium dodecyl sulfate (SDS, 8.1%). Samples were heated at 95°C during 30 min, and after cooling by 10 min, 100 *μ*L of Milli-Q water and 500 *μ*L of n-butanol were added. After centrifugation (3,000 ×g during 10 min at 15°C), the organic phase (150 *μ*L) was placed in a microplate reader, and the fluorescence registered after excitation at 520 nm and emission of 580 nm. The concentration of TBARS (nmol/mg of wet tissue) was calculated employing tetramethoxypropane (TMP) as standard.

### 2.11. Protein Determination

Protein was measured by the Coomassie blue method [36] using bovine serum albumin as standard.

### 2.12. Statistical Analysis

AChE activity and antioxidant analyses were expressed as means ± S.E.M. and analyzed by one-way analysis of variance (ANOVA). Post hoc comparisons were made using Tukey's test and orthogonal comparisons. Before ANOVA, its assumptions (normality and variances homogeneity) were checked. Molecular data were expressed as means ± S.E.M. and analyzed by Student's *t*-test. In every case, the significance level was fixed in 5% (*α* = 0.05).

## 3. Results

### 3.1. C_60_ Suspensions of Nano/Microparticles

Although DMSO is known to show low toxicity by itself [24, 37], appropriate experimental controls must be employed to eliminate its influence. In this study, the DMSO was diluted to result in 12.5% DMSO as a final concentration. Any signal of toxicity, that is, mortality or even transient alterations in behavior, was observed in the vehicle control group (12.5% DMSO). In addition, control group (saline) and vehicle control group were never statistically different in the conditions tested.

**Figure 1:**
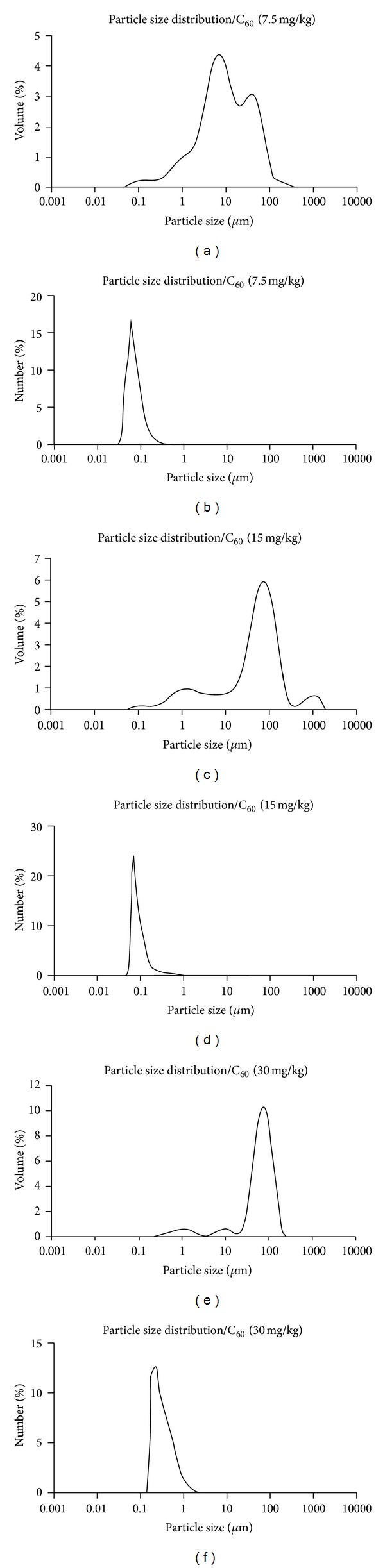
Size distribution in suspensions of nano/microparticles. Mean diameters distribution of fullerene C_60_ suspensions (7.5, 15, and 30) was determined over the volume ((a), (c), and (e)) and over the number ((b), (d), and (f)) of the nano/microparticles.

The nano/microparticles mean diameters over the volume showed wide distributions (60 nm–316 *μ*m; 69 nm–1,905 *μ*m; 182 nm–208 *μ*m, for suspensions 7.5, 15, and 30, resp.), with most abundant sizes in the micrometric range (Figures 1(a), 1(c), and 1(e)). The SPAN values increased with the dilution of suspensions (5.843, 3.003, and 1.607 to suspensions 7.5, 15, and 30, resp.), showing a narrow size distribution with lower dilutions. On the other hand, it is important to emphasize that when considering the mean diameters over the number distribution, we observed that the greater part of particles were under nanometric sizes (Figures 1(b), 1(d), and 1(f)).

### 3.2. Acetylcholinesterase Enzymatic Activity and Gene Expression

**Figure 2:**
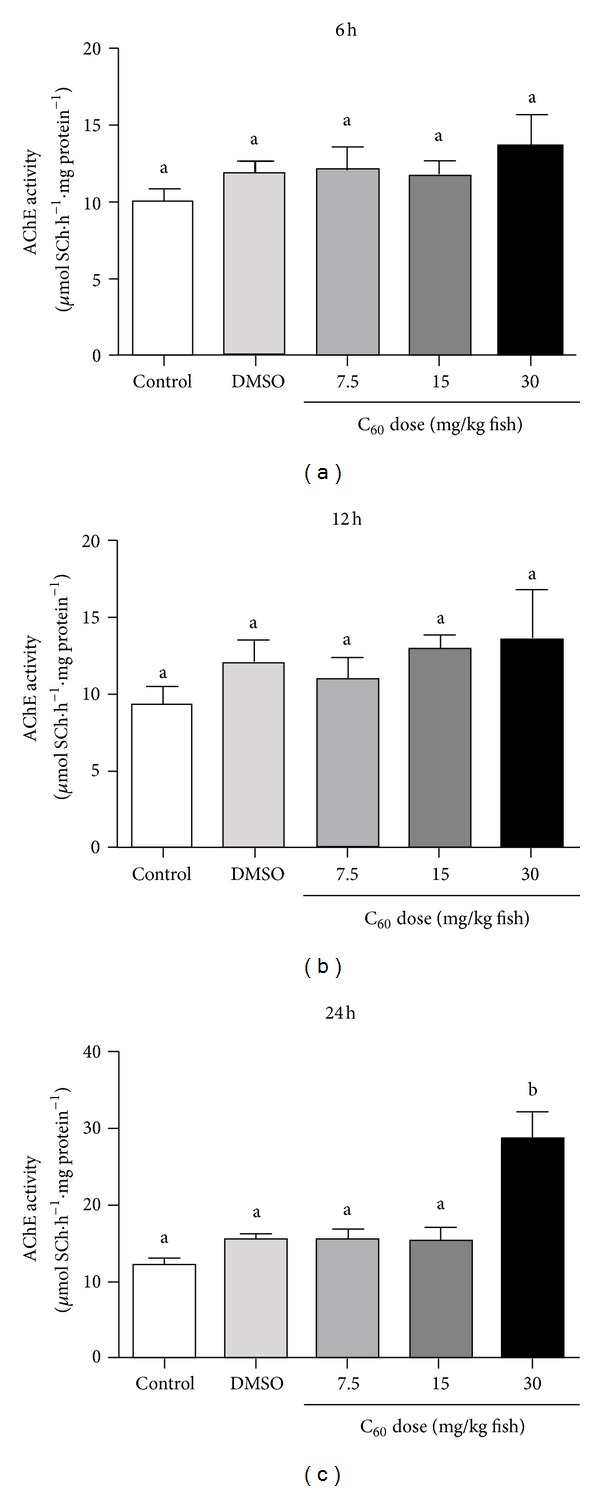
*In vivo* AChE activity in zebrafish brain after 06 (a), 12 (b), and 24 hours (c) of fullerene C_60_ exposure at distinct concentrations (7.5–30 mg/kg fish). Bars represent the mean ± SEM of at least three different experiments, each one performed in triplicate. The specific enzyme activity is reported as micromoles of thiocholine released per hour per milligram of protein. Bars represent the mean ± SEM of at least three independent experiments, each one performed in triplicate. Different letters indicate significant differences (*P* < 0.05) between groups.

**Figure 3:**
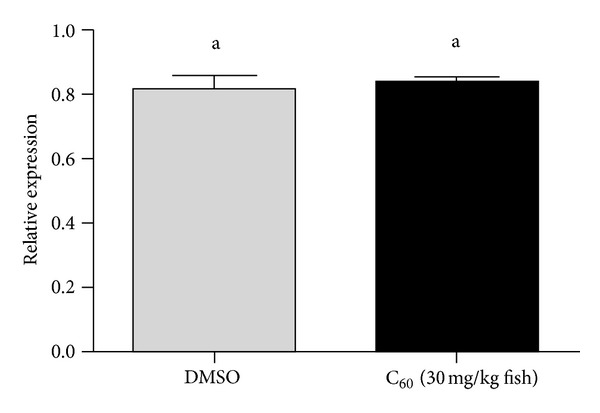
RT-qPCR analysis. Relative *ache* expression profile after fullerene C_60_ exposure (30 mg/kg fish for 24 hours) on zebrafish brain. Bars represent the mean ± SEM.

The effect of different C_60_ concentrations and times of exposure on brain AChE activity was demonstrated by performing (*in vivo*) experiments using adult zebrafish. None of the concentrations tested (7.5, 15, and 30 mg/kg) altered AChE activity when zebrafish were exposed to C_60_ during 6 hours (Figure 2(a)) and 12 hours (Figure 2(b)). However, the analysis for 24 hours demonstrated that animals treated with the concentration of 30 mg/kg presented a significant increase in AChE activity (28.54 ± 3.72 *μ*mol SCh·h^−1^·mg protein^−1^; *P* = 0.0001) when compared to saline (12.19 ± 0.55 *μ*mol SCh·h^−1^·mg protein^−1^; *P* = 0.0001) and to the vehicle control group (15.46 ± 0.57 *μ*mol SCh·h^−1^·mg protein^−1^; *P* = 0.0001) (Figure 2(c)). The upregulation of brain AChE activity after exposure to C_60_ (30 mg/kg for 24 hours) could be a consequence of transcriptional control. In order to determine if transcriptional regulation of AChE gene has occurred, a RT-qPCR analysis was performed. The results have shown that AChE transcript levels were not enhanced when compared to the vehicle control group (*P* = 0.6695; Figure 3) suggesting that the activation of brain AChE is not directly related with the transcriptional control.

### 3.3. *In  Vitro* Effects of C_60_ Suspensions on Acetylcholinesterase Activity

**Figure 4:**
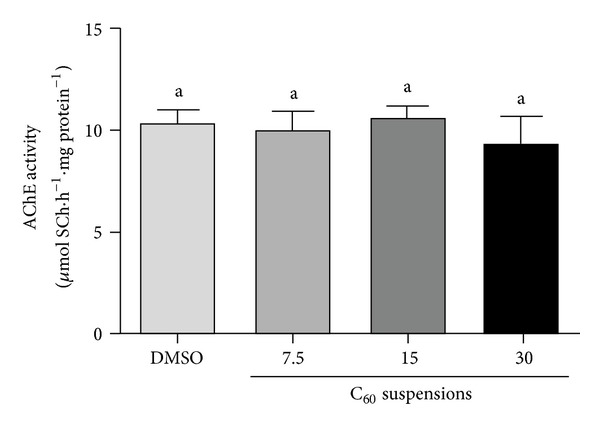
*In vitro* AChE activity. *In vitro* effect of different C_60_ suspensions (7.5, 15, and 30) on ACh hydrolysis in zebrafish brain. Bars represent the mean ± SEM of at least three different experiments, each one performed in triplicate.

To verify whether C_60_ nano/microparticles might have a direct effect on the enzyme, we tested the *in vitro* effect of C_60_ suspensions on AChE activity in zebrafish brain. The results showed that C_60_ suspensions did not bring about any alteration in AChE activity (*P* = 0.7701; Figure 4).

### 3.4. Antioxidant Analysis

**Figure 5:**
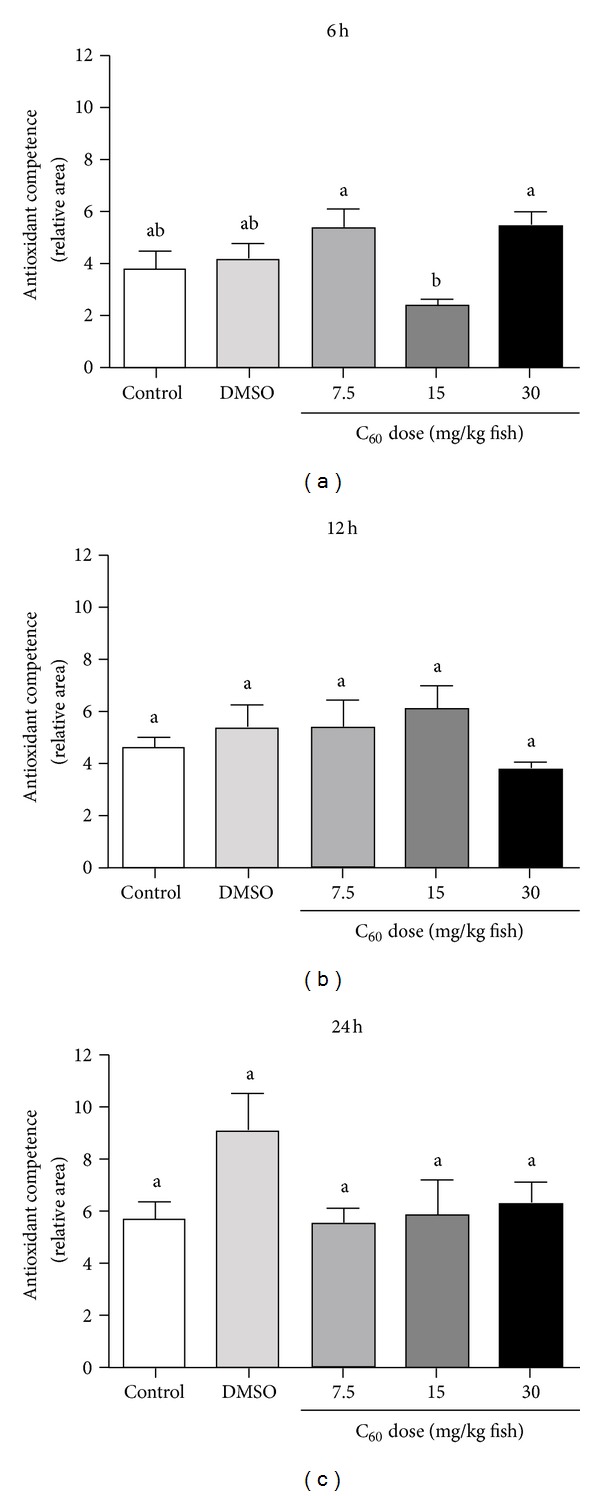
Antioxidant capacity. Total antioxidant capacity against peroxyl radical in zebrafish brain after 06 (a), 12 (b), and 24 hours (c) of fullerene C_60_ exposure at distinct concentrations (7.5–30 mg/kg fish). Bars represent the mean ± SEM of at least three independent experiments. Different letters indicate significant differences (*P* < 0.05) between groups.

**Figure 6:**
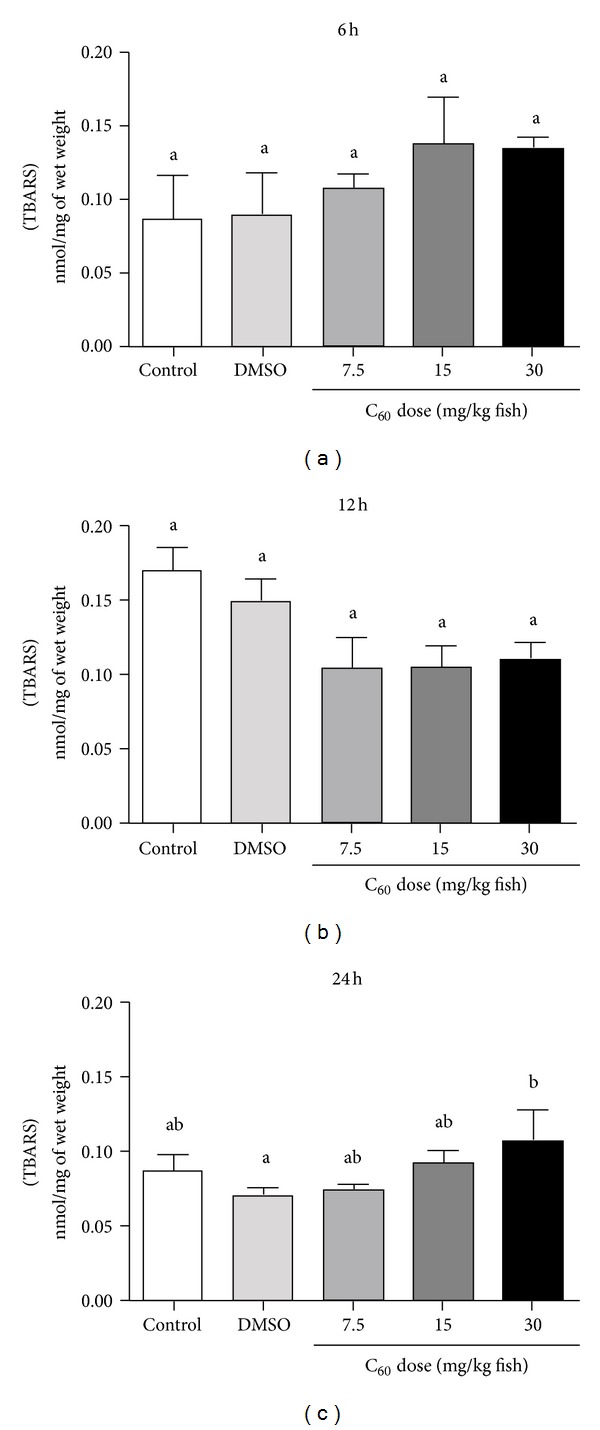
Lipid oxidative damage. Concentration of thiobarbituric acid reactive substances (TBARS; nmol/mg of wet weight) in zebrafish brain after 06 (a), 12 (b) and 24 hours (c) of fullerene C_60_ exposure at distinct concentrations (7.5–30 mg/kg fish). Bars represent the mean ± SEM of at least three independent experiments. Different letters indicate significant differences (*P* < 0.05) between groups.

The total antioxidant competence against peroxyl radicals showed an augmented response (lower relative area) in brains of zebrafish exposed to C_60_ (15 mg/kg) for 6 hours when compared to zebrafish exposed to C_60_ (7.5 and 30 mg/kg) for 6 hours (*P* = 0.0209; Figure 5(a)). No other differences were observed under the experimental conditions (Figures 5(b) and 5(c)). Oxidative damage, measured by lipid peroxidation (TBARS assays), showed a pro-oxidant condition elicited by C_60_ at the highest dose (30 mg/kg) after 24 hours (*P* = 0.0194; Figures 6(a), 6(b), and 6(c)).

## 4. Discussion

Although technologies evolving nano/microparticles have considerable potential in diverse applications, it is crucial to determine possible toxicity of these technological products before extensive use. Little is known about the toxic effects of fullerenes in brain. At present, only few studies presenting contradictory findings have evaluated possible neurotoxic effects of fullerenes exposure. For instance, it was already suggested that C_60_ did not cross the blood-brain barrier [38], whereas the results obtained by Mokrushin [39] suggested that fullerenes possess marked neurotropic properties and are neurotoxic substances irreversibly blocking the electrical activity of the nervous tissue.

Neurotoxicity of C_60_ in fish species has been previously reported [11, 40, 41]. Generation of reactive oxygen species (ROS) by C_60_ is influenced in part by the presence and type of illumination due to the photoexcitation of C_60_ by UV and visible light [42] or even to by-products of the organic solvents employed to prepare C_60_ suspensions [40]. For this reason, the C_60_ suspensions were prepared under the protection of light, and i.p. injection was adopted as administration route in the *in vivo* protocols, an experimental condition that avoids the influence of light in the analyzed variables. Also, *in vitro* assays were run in darkness.

The characterization of the size and stability of C_60_ nanoparticles in suspension is very important to evaluate their toxicity once particle size can change during the preparation of the suspension, dilution, and exposure [11]. In this study, the nano/microparticles mean diameters over the volume in the C_60_ suspensions showed wide distributions with most abundant sizes in the micrometric range. The C_60_ has a tendency to form aggregates very easily [7], and this may be a possible cause of this wide distribution. In contrast, the nano/microparticles mean diameters over the number of distribution in the C_60_ suspensions demonstrated that the greater part of particles was under nanometric sizes. Totsuka and colleagues [43] also observed wide distributions by dynamic light scattering in formulations manufactured with C_60_. Take into account that particle sizes limit their ability to translocate to different tissues [3, 44] the assessment of the distribution of C_60_ material on zebrafish brain would be useful to reinforce our findings. Nevertheless, studies have demonstrated that particles even larger than the nano/microparticles of this study were able to reach brain. For instance, the study of Sarlo and colleagues [45] showed that 1000 nm latex fluorospheres were recovered from rat brains one and 24 hours after intravenous (i.v.) injection. In addition, the study by Zhu and colleagues [41] showed that exposure to fullerene aggregates suspended in water (with average diameters of approximately 349 and/or 1,394 nm) decreased glutathione in brain of juvenile carps (*Carassius auratus*). 

In the present study, we have evaluated the effect of different C_60_ doses (7.5, 15, and 30 mg/kg) and different times of exposure (6, 12, and 24 hours) on AChE activity and *ache* expression in zebrafish brain. In the concentrations tested, only the animals exposed to 30 mg/kg for 24 hours have shown enhanced AChE activity. The RT-qPCR results suggested that the activation of brain AChE is not directly related with the transcriptional control. The *in vitro* results indicated that none of the C_60_ suspensions had a direct effect on the enzyme. It is important to highlight that *in vitro* experiments do not evaluate the influence of other indirect mechanisms such as cell signaling pathways. Altogether, our results indicate that the effect of intraperitoneal exposure to nano/microparticles of fullerene (C_60_) on brain AChE activity was neither related to AChE gene activation nor to the direct action of this molecular material on this enzyme but probably involved a posttranscriptional or posttranslational modulation of this enzymatic activity.

Moreover, we have shown the effects of C_60_ exposure over the antioxidant competence and lipid peroxidation in zebrafish brain. The results demonstrated that the exposition to 30 mg/kg during 24 hours did not alter the antioxidant competence and yielded in higher levels of lipid peroxidation. The lack in the antioxidant response to a pro-oxidant situation could explain the augmented lipid peroxidation found in zebrafish brain. In addition, Totsuka and colleagues [43] reported increased micronuclei frequencies, induced DNA damage, and increased mutant frequencies after C_60_ nano/microparticles suspension exposure.

AChE is indispensable for terminating acetylcholine-mediated neurotransmission at cholinergic synapses [46]. In this context, AChE is inhibited by organophosphorus and carbamate insecticides and by neurotoxins, which are structural analogues of acetylcholine [47]. In addition, there are lines of evidence to suggest that AChE contributes to diverse physiological processes through its involvement in the regulation of cell proliferation, differentiation, and survival. As a consequence, more recently AChE has been redefined as an important regulator of apoptosis, because it can be induced by a variety of apoptotic stimuli [48, 49]. It is well known that apoptosis underlies the neurotoxic effects of various compounds. Moreover, zebrafish brain AChE activation has also been demonstrated as a consequence of exposure to known neurotoxic compounds, including aluminum [20] and ethanol [21] and to the cyanobacterial toxin microcystin-LR [50].

The antioxidant or pro-oxidant effects induced by C_60_ exposure are still a debatable issue [40, 51]. C_60_ is photoexcited under UV or visible light [42], a condition, for example, that elicited lipid peroxidation in brains of common carp (*C. carpio*) [11]. On the other hand, the absence of light did not completely inhibit oxidative stress generation in embryonic zebrafish after C_60_ exposure [52]. Moreover, cell membrane lipid peroxidation was suggested as the main mechanism of toxicity caused by fullerenes' exposure [10, 11]. In addition, the study by Sayes and colleagues [9] reported cytotoxicity (cell apoptosis) in three human cell cultures including astrocytes caused by cell membrane lipid peroxidation due to exposure to nano-C_60_ (a water-soluble fullerene species). 

The results presented in this paper provide further experimental evidence that C_60_ exposure can be neurotoxic. Adult zebrafish exposed to nonphotoexcited C_60_ nano/microparticles (30 mg/kg for 24 hours) have shown enhanced AChE activity and augmented lipid peroxidation in brain. The study by Melo and colleagues [53] proposed a mechanism to explain enhanced AChE activity mediated by oxidative stress generation in cultured retinal cells exposed to amyloidogenic peptide A*β*_25–35_. The incubation with A*β*_25–35_ led to an increment of ROS formation and increased significantly lipid peroxidation levels which decreased cell membrane order and ultimately led to the exposure of more active sites of the AChE. As a consequence, AChE activity was increased. It is possible to speculate that similar events had occurred in zebrafish brain. However, further studies must be performed in order to evaluate this hypothesis.

## Figures and Tables

**Table 1:** PCR primers design.

Proteins	Primer sequences (5′-3′)	Accession number (mRNA)
EF1α*	F: CTGGAGGCCAGCTCAAACAT R: ATCAAGAAGAGTAGTACCGCTAGCATTAC	NSDART00000023156
Rpl13α*	F: TCTGGAGGACTGTAAGAGGTATGC R: AGACGCACAATCTTGAGAGCAG	NM_212784
*ac* he**	F: GCTAATGAGCAAAAGCATGTGGGCTTG R: TATCTGTGATGTTAAGCAGACGAGGCAGG	NM_131846

*According to Tang et al. [22].

**Designed by the authors.

## Abbreviations

ChAT: Choline acetyltransferase
ACh: Acetyl choline
AChE: Acetylcholinesterase
BuChE: Butyrylcholinesterase
UV: Ultraviolet light
ACAP: Antioxidant capacity
TBARS: Thiobarbituric acid reactive substances
EDTA: Ethylenedioxy-diethylene-dinitrilo-tetraacetic acid
EGTA: Ethylene glycol bis(beta amino ethylether)-N,N,N′,N′-tetraacetic acid
DTNB: 5,5′-Dithiobis-2-nitrobenzoic acid
HEPES: 4-(2-Hydroxyethyl)-1-piperazineethanesulfonic acid
BHT: Butylated hydroxytoluene
ABAP: 2,2′-Azobis(2-methylpropionamidine) dihydrochloride
KCl: Potassium chloride
SDS: Sodium dodecyl sulfate
TMP: Tetramethoxypropane
DMSO: Dimethyl sulfoxide
MeOH: Methanol
ACSCh: Acetylthiocholinex hydrolysis
SCh: Thiocholine
ROS: Reactive oxygen species.

## References

[1] ISO/TC 229 http://www.iso.org/iso/iso_technical_committee?commid=381983.

[2] Project on Emerging Nanotechnologies http://www.nanotechproject.org/inventories/consumer.

[3] Oberdörster G, Oberdörster E, Oberdörster J (2005). Nanotoxicology: an emerging discipline evolving from studies of ultrafine particles. *Environmental Health Perspectives*.

[4] Kahru A, Dubourguier H (2010). From ecotoxicology to nanoecotoxicology. *Toxicology*.

[5] Kroto HW, Heath JR, O’Brien SC, Curl RF, Smalley RE (1985). C_60_: buckminsterfullerene. *Nature*.

[6] Prato M (1997). [60]Fullerene chemistry for materials science applications. *Journal of Materials Chemistry*.

[7] Montellano A, Da Ros T, Bianco A, Prato M (2011). Fullerene C_60_ as a multifunctional system for drug and gene delivery. *Nanoscale*.

[8] Kolosnjaj J, Szwarc H, Moussa F (2007). Toxicity studies of fullerenes and derivatives. *Advances in Experimental Medicine and Biology*.

[9] Sayes CM, Gobin AM, Ausman KD, Mendez J, West JL, Colvin VL (2005). Nano-C_60_ cytotoxicity is due to lipid peroxidation. *Biomaterials*.

[10] Oberdörster E (2004). Manufactured nanomaterials (fullerenes, C_60_) induce oxidative stress in the brain of juvenile largemouth bass. *Environmental Health Perspectives*.

[11] Shinohara N, Matsumoio T, Gamo M (2009). Is lipid peroxidation induced by the aqueous suspension of fullerene C_60_ nanoparticles in the brains of *Cyprinus carpio*?. *Environmental Science and Technology*.

[12] Soreq H, Seidman S (2001). Acetylcholinesterase—new roles for an old actor. *Nature Reviews Neuroscience*.

[13] Hernández PP, Allende ML (2008). Zebrafish (*Danio rerio*) as a model for studying the genetic basis of copper toxicity, deficiency, and metabolism. *The American Journal of Clinical Nutrition*.

[14] Bertrand C, Chatonnet A, Takke C (2001). Zebrafish acetylcholinesterase is encoded by a single gene localized on linkage group 7. Gene structure and polymorphism; molecular forms and expression pattern during development. *The Journal of Biological Chemistry*.

[15] van Dyk JS, Pletschke B (2011). Review on the use of enzymes for the detection of organochlorine, organophosphate and carbamate pesticides in the environment. *Chemosphere*.

[16] Rico EP, Rosemberg DB, Senger MR (2006). Methanol alters ecto-nucleotidases and acetylcholinesterase in zebrafish brain. *Neurotoxicology and Teratology*.

[17] Richetti SK, Rosemberg DB, Ventura-Lima J, Monserrat JM, Bogo MR, Bonan CD (2011). Acetylcholinesterase activity and antioxidant capacity of zebrafish brain is altered by heavy metal exposure. *NeuroToxicology*.

[18] Monserrat JM, Yunes JS, Bianchini A (2001). Effects of *Anabaena spiroides* (cyanobacteria) aqueous extracts on the acetylcholinesterase activity of aquatic species. *Environmental Toxicology and Chemistry*.

[19] Pereira VM, Bortolotto JW, Kist LW (2012). Endosulfan exposure inhibits brain AChE activity and impairs swimming performance in adult zebrafish (*Danio rerio*). *NeuroToxicology*.

[20] Senger MR, Seibt KJ, Ghisleni GC, Dias RD, Bogo MR, Bonan CD (2011). Aluminum exposure alters behavioral parameters and increases acetylcholinesterase activity in zebrafish (*Danio rerio*) brain. *Cell Biology and Toxicology*.

[21] Rico EP, Rosemberg DB, Dias RD, Bogo MR, Bonan CD (2007). Ethanol alters acetylcholinesterase activity and gene expression in zebrafish brain. *Toxicology Letters*.

[22] Tang R, Dodd A, Lai D, McNabb WC, Love DR (2007). Validation of zebrafish (*Danio rerio*) reference genes for quantitative real-time RT-PCR normalization. *Acta Biochimica et Biophysica Sinica*.

[23] Isaacson CW, Usenko CY, Tanguay RL, Field JA (2007). Quantification of fullerenes by LC/ESI-MS and its application to *in vivo* toxicity assays. *Analytical Chemistry*.

[24] Kim KT, Jang MH, Kim JY, Kim SD (2010). Effect of preparation methods on toxicity of fullerene water suspensions to Japanese medaka embryos. *Science of the Total Environment*.

[25] Ji Z, Zhang D, Li L (2009). The hepatotoxicity of multi-walled carbon nanotubes in mice. *Nanotechnology*.

[26] Zhang D, Deng X, Ji Z (2010). Long-term hepatotoxicity of polyethylene-glycol functionalized multi-walled carbon nanotubes in mice. *Nanotechnology*.

[27] Clichici S, Mocan T, Filip A (2011). Blood oxidative stress generation after intraperitoneal administration of functionalized single-walled carbon nanotubes in rats. *Acta Physiologica Hungarica*.

[28] Kusters KA, Wijers JG, Thoenes D (1991). Particle sizing by laser diffraction spectrometry in the anomalous regime. *Applied Optics*.

[29] Phelps HA, Runft DL, Neely MN (2009). Adult zebrafish model of streptococcal infection. *Current Protocols in Microbiology*.

[30] da Rocha AM, Ferreira JR, Barros DM (2013). Gene expression and biochemical responses in brain of zebrafish *Danio rerio* exposed to organic nanomaterials: carbon nanotubes (SWCNT) and fullerenol (C_60_(OH)_18-22_(OK_4_)). *Comparative Biochemistry and Physiology A*.

[31] Seibt KJ, Oliveira RDL, Rico EP, Dias RD, Bogo MR, Bonan CD (2009). Antipsychotic drugs inhibit nucleotide hydrolysis in zebrafish (*Danio rerio*) brain membranes. *Toxicology In Vitro*.

[32] Siebel AM, Rico EP, Capiotti KM (2010). *In vitro* effects of antiepileptic drugs on acetylcholinesterase and ectonucleotidase activities in zebrafish (*Danio rerio*) brain. *Toxicology In Vitro*.

[33] Ellman GL, Courtney KD, Andres V, Featherstone RM (1961). A new and rapid colorimetric determination of acetylcholinesterase activity. *Biochemical Pharmacology*.

[34] Amado LL, Garcia ML, Ramos PB (2009). A method to measure total antioxidant capacity against peroxyl radicals in aquatic organisms: application to evaluate microcystins toxicity. *Science of the Total Environment*.

[35] Oakes KD, van der Kraak GJ (2003). Utility of the TBARS assay in detecting oxidative stress in white sucker (*Catostomus commersoni*) populations exposed to pulp mill effluent. *Aquatic Toxicology*.

[36] Bradford MM (1976). A rapid and sensitive method for the quantitation of microgram quantities of protein utilizing the principle of protein dye binding. *Analytical Biochemistry*.

[37] Rubin LF (1983). Toxicologic update of dimethyl sulfoxide. *Annals of the New York Academy of Sciences*.

[38] Yamada T, Jung D, Sawada R, Matsuoka A, Nakaoka R, Tsuchiya T (2008). Effects intracerebral microinjection and intraperitoneal injection of [60]Fullerene on brain functions differ in rats. *Journal of Nanoscience and Nanotechnology*.

[39] Mokrushin AA (2001). Neurotoxic effects of fullerenes on the electrical activity of surviving sections of the rat brain olfactory cortex. *Doklady Biological Sciences*.

[40] Henry TB, Menn F, Fleming JT, Wilgus J, Compton RN, Sayler GS (2007). Attributing effects of aqueous C_60_ nano-aggregates to tetrahydrofuran decomposition products in larval zebrafish by assessment of gene expression. *Environmental Health Perspectives*.

[41] Zhu X, Zhu L, Lang Y, Chen Y (2008). Oxidative stress and growth inhibition in the freshwater fish *Carassius auratus* induced by chronic exposure to sublethal fullerene aggregates. *Environmental Toxicology and Chemistry*.

[42] Kamat JP, Devasagayam TPA, Priyadarsini KI, Mohan H (2000). Reactive oxygen species mediated membrane damage induced by fullerene derivatives and its possible biological implications. *Toxicology*.

[43] Totsuka Y, Higuchi T, Imai T (2009). Genotoxicity of nano/microparticles in *in vitro* micronuclei, *in vivo* comet and mutation assay systems. *Particle and Fibre Toxicology*.

[44] Jani P, Halbert GW, Langridge J, Florence AT (1990). Nanoparticle uptake by the rat gastrointestinal mucosa: quantitation and particle size dependency. *Journal of Pharmacy and Pharmacology*.

[45] Sarlo K, Blackburn KL, Clark ED (2009). Tissue distribution of 20 nm, 100 nm and 1000 nm fluorescent polystyrene latex nanospheres following acute systemic or acute and repeat airway exposure in the rat. *Toxicology*.

[46] Taylor P, Radić Z (1994). The cholinesterases: from genes to proteins. *Annual Review of Pharmacology and Toxicology*.

[47] Matsumura F (1985). *Toxicology of Insecticides*.

[48] Zhang XJ, Yang L, Zhao Q (2002). Induction of acetylcholinesterase expression during apoptosis in various cell types. *Cell Death and Differentiation*.

[49] Jiang H, Zhang XJ (2008). Acetylcholinesterase and apoptosis: a novel perspective for an old enzyme. *FEBS Journal*.

[50] Kist LW, Rosemberg DB, Pereira TCB (2012). Microcystin-LR acute exposure increases AChE activity via transcriptional ache activation in zebrafish (*Danio rerio*) brain. *Comparative Biochemistry and Physiology C*.

[51] Henry TB, Petersen EJ, Compton RN (2011). Aqueous fullerene aggregates (nC_60_) generate minimal reactive oxygen species and are of low toxicity in fish: a revision of previous reports. *Current Opinion in Biotechnology*.

[52] Usenko CY, Harper SL, Tanguay RL (2008). Fullerene C_60_ exposure elicits an oxidative stress response in embryonic zebrafish. *Toxicology and Applied Pharmacology*.

[53] Melo JB, Agostinho P, Oliveira CR (2003). Involvement of oxidative stress in the enhancement of acetylcholinesterase activity induced by amyloid beta-peptide. *Neuroscience Research*.

